# Male Mating Preference for Larger Females Does Not Vary Among Age Classes in the Long‐Lived Beetle *Bolitotherus cornutus*

**DOI:** 10.1002/ece3.71958

**Published:** 2025-08-14

**Authors:** Griffin M. Jiron, Charlotte A. Greene, Edmund D. Brodie, Vincent A. Formica

**Affiliations:** ^1^ Mountain Lake Biological Station and Department of Biology University of Virginia Charlottesville Virginia USA; ^2^ Department of Biology Swarthmore College Swarthmore Pennsylvania USA

## Abstract

Theory and past experimental work suggest that as males age, the strength of their mate preference should decrease. However, the empirical work investigating this question has primarily been conducted in insects that have very short life spans and often live for just a single mating season. This leaves a gap in our understanding of the relationship between male mate preference and age across taxa, as age can conflate with other ecological changes in a single mating season. In this study, we ask how the strength of preference for large female body size changes as males age in a long‐lived insect, *Bolitotherus cornutus*. We used a two‐pronged approach of both laboratory behavior trials and cross‐sectional analyses of observations in a wild metapopulation to answer this question. We found that males overall exhibited a preference for large females, but there was no significant difference between the preference strength of young and old males in either the laboratory experiment or field observations. Our work suggests that age may not play as important a role in variation in male mate preference as predicted by previous findings, especially in long‐lived animals. Instead, processes such as senescence, breeding season termination, or mate availability may be stronger drivers of male mate preference variation.

## Graphical abstract

An increasing amount of research has found that males can also be selective for female traits. Surprisingly, old males in the long‐lived beetle *B. cornutus* were not significantly less choosy than young males. This calls attention to our assumptions about aging and the trends in behavior it may drive.

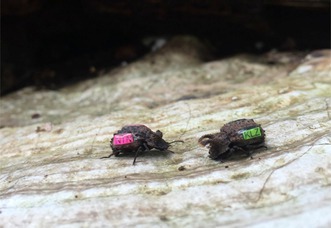

## 1 Introduction

Despite a historical perspective of “choosy females” and “indiscriminate males” (Darwin 1871), substantial evidence supports the idea that males can similarly have a preference for females, even in systems where males invest no more than sperm (Dewsbury 1982; Edward and Chapman 2011). Male mate preference has been predicted in mathematical models (Johnstone et al. 1996; Servedio and Lande 2006) and observed empirically across taxa, including insects (Bateman and Fleming 2006; Chenoweth et al. 2007; Formica et al. 2016), arachnids (Bel‐Venner et al. 2007; Baruffaldi and Andrade 2015; Rundus et al. 2015), crustaceans (Goshimaa et al. 1996; Reading and Backwell 2007), fish (Amundsen and Forsgren 2001; Candolin and Salesto 2009), reptiles (Swierk et al. 2013), birds (Griggio et al. 2009; Amundsen et al. 1997), and mammals (Craig et al. 2002), including the closest extant relative of humans, chimpanzees (Muller et al. 2006). Male mate preference is most likely to evolve when the relative costs of reproduction are high, when males frequently encounter potential mates, and when females are varied in quality, often as a measure of fecundity or mating status (Edward and Chapman 2011). Under these conditions, males are predicted to gain a reproductive benefit from strategically allocating their mating effort and sperm, giving rise to the evolution of mate preference (Edward and Chapman 2011).

Although these conditions have been well‐described theoretically and experimentally, one influence on male mate preference evolution that has received comparatively less attention is male age (but see Engqvist and Sauer 2002; Dhole and Pfennig 2014; Baxter et al. 2015; Rundus et al. 2015). Ample life‐history research has shown that age is strongly associated with changes in male reproductive behaviors, including courtship duration and intensity (Aich et al. 2021; Karl et al. 2013; Mysterud et al. 2005; Jones and Elgar 2004), mate searching (De Luca and Cocroft 2011), and even behaviors important for male–male competition (Trumbo 2012). Additionally, the relationship between age and mate preference has been thoroughly investigated in female choice, in which females often demonstrate weaker preferences for male traits as they age (Moore and Moore 2001). This pattern emerges because as females age, their opportunities for reproduction decrease, and the costs of losing mating opportunities outweigh the potential benefits of reserving their mating events for high quality males (Jennions and Petrie 1997). However, the relationship between male age and male preference for female traits remains far less studied.

Theoretical models of male mating behavior predict that as time passes and future opportunities to mate decrease, a male would benefit from subsequently decreasing his level of choosiness (Galvani and Johnstone 1998). This trend has indeed been observed over the course of a breeding cycle in short‐lived insects and arachnids, in which previously choosy males become indiscriminate as their breeding window comes to an end (Engqvist and Sauer 2002; Dhole and Pfennig 2014; Baxter et al. 2015; Rundus et al. 2015). However, these studies on male age influencing male mating preference remain limited to systems that live for one breeding cycle, making it difficult to separate the short‐term effects of an ending breeding season from the longer‐term effects of male age. Many systems that exhibit male mate preference also live for multiple breeding seasons (Griggio et al. 2009; Amundsen et al. 1997; Reading and Backwell 2007; Craig et al. 2002; Muller et al. 2006); investigating the strength of preference among age groups in a long‐lived species would therefore allow for a more accurate understanding of how aging may influence this behavior in populations with diverse age structures. Understanding how male age may influence male mate preference may also further reveal how female traits evolve under sexual selection in age‐structured populations.

In this study, we investigated the effect of male age on male mate preference in the forked fungus beetle (*Bolitotherus cornutus*). This system is well suited for investigating male preference and age, as males frequently encounter females and have an extensive courtship routine that limits their ability to mate with multiple females simultaneously (Conner 1989). Additionally, males live for multiple years and show a preference for large female body size, a known predictor of variation in fecundity (Conner 1989; Formica et al. 2016). Our study used a two‐pronged approach of field and laboratory methods. We performed a laboratory experiment consisting of a series of no‐choice mating trials to capture fine‐scaled differences in male courtship behavior that could indicate mate preference for female size. This laboratory experiment allowed us to directly manipulate male age and female size and provides a rigorous test of male mating preference that is often difficult to capture through natural observations. As Barry and Kokko propose in their 2010 model, however, placing individuals in novel ecological contexts may subsequently spawn novel behaviors, such as mate preference in males. We accounted for this by performing a multi‐year cross‐sectional study in a large metapopulation of *B. cornutus* to track differences in male preferences for female size in the wild. In alignment with past theoretical (Galvani and Johnstone 1998; Williams 1966) and empirical work (Engqvist and Sauer 2002; Dhole and Pfennig 2014; Baxter et al. 2015; Rundus et al. 2015), we predicted that young males would show a stronger preference for larger, more fecund females than older males, who would be more indiscriminate.

## 2 Methods

### 2.1 Study System

**FIGURE 1:**
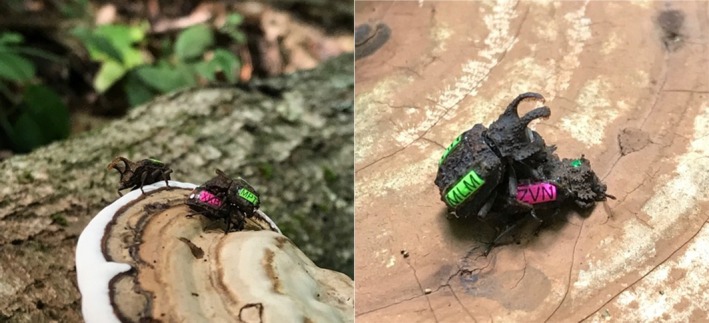
*Bolitotherus cornutus* mating behaviors in the wild. Male (green labels) forked fungus beetles perform a suite of observable mating behaviors, including the “waggle” dance (left) and attempting copulation (right). Picture credits belong to Vince Formica.

The forked fungus beetle (*Bolitotherus cornutus*) is a long‐lived beetle species native to eastern North America (Liles 1956; Pace 1967; Whitlock 1992); the beetles live, eat, and reproduce on three main fungus species growing on decaying deciduous trees: *Ganoderma tsugae*, *Ganoderma applanatum*, and *Fomes fomentarius* (Liles 1956). They lay eggs, mate, and perform all social interactions on or near these brackets; larvae then feed and pupate within the fungal fruiting bodies of these brackets. As a result, we are able to observe all mating and social behaviors in the wild (Figure 1). The beetles are also easily captured and marked, allowing us to track wild populations across multiple years and investigate questions regarding age‐related behaviors in both the lab and field. The beetles are also sexually dimorphic—males typically possess prominent thoracic horns with distinct orange hairs as well as fuzzy patches of orange hair on their underside, both of which are absent in females (Liles 1956; Pace 1967).

Forked fungus beetles are an ideal system to study male choosiness because previous work suggests that males largely control whether courtship is initiated (Conner 1988). This courtship behavior is also easily observable to the naked eye, where males will mount a female, aligned so that his posterior is above her head (Figure 1), and periodically “waggle” to court her (Conner 1989). The male intermittently interrupts his courtship to attempt copulation by reorienting himself so that his head is directly above hers and his genitalia are in contact with hers (Figure 1). It appears that the female does not control whether the male attempts copulation. She does, however, have full control over whether the copulation attempt is successful. To accept a copulation attempt, the female opens her anal sternite, allowing the male's aedeagus to enter, and the male to transfer a large spermatophore. If the male is successful in inseminating the female, he will remount her, with his head over her head, and guard her from other males that may try to court or copulate with her (Conner 1989). Laboratory experiments have shown that this guarding behavior is a reliable indication of a successful spermatophore transfer (Conner 1989). If unsuccessful in copulating with the female, the male will often return to the courting position and resume waggling. These bouts of courtship and guarding often last upwards of 10 h, imposing a significant time and energy investment on males for each mating event (Conner 1989; Formica et al. 2016; Greene et al. 2025).

Previous work in this system demonstrated male choosiness for larger and more fecund females both in the wild and in laboratory settings (Formica et al. 2016). However, this study did not investigate age, as the ages of males in the population were unknown. *B. cornutus* are holometabolous insects, and their body size does not change after they eclose, allowing us to independently investigate age and size‐based preferences in this system.

### 2.2 Laboratory Experiment

Because *B. cornutus* mating takes place through sequential mate choice, we opted for a no‐choice laboratory experiment, in which a male was placed in a mating trial with a single female. We used this methodological approach as opposed to a commonly used two‐choice approach to avoid potential bias in detecting choice (Barry and Kokko 2010; Dougherty and Shuker 2015). Additionally, no‐choice trials both remove potential confounds of intrasexual interactions during the behavior trial and test the absolute preference males have for large female body size (Dougherty 2020), allowing us a more rigorous testing environment than we can observe in natural populations.

#### 2.2.1 Beetle Collection and Husbandry for Laboratory Experiment

We collected all beetles of known age used for this experiment in June 2024—near the start of the beetles' breeding season—from the Mountain Lake Biological Station breeding enclosures (“beetlearies”), a collection of semi‐natural enclosures with self‐contained captive breeding *B. cornutus* populations. The methods for construction and maintenance of these breeding populations are described in Cook et al. (2023), in which the beetlearies were founded with wild beetles collected in 2017 that were not found in the 16 major subpopulations used for the field observations. We created 12 screened‐in enclosures and placed 36 beetles in each and allowed beetles to breed and deposit eggs on the fungus brackets in the enclosure. All beetles placed in the beetlearies in 2017 were marked with a unique color with the same enamel paint used in the wild populations. Every year for the next 6 years, we searched the beetlearies for newly emerged beetles. Any beetle that did not have a paint color on its elytra was assumed to be a one‐year‐old beetle. Each year, adults were shuffled between enclosures to prevent inbreeding depression and mimic natural dispersal and migration conditions.

After collection, we housed beetles in individual cups with perforated lids for at least 10 days in a temperature‐controlled room at 22°C with an 18:6 light:dark cycle. Each cup contained wet mulch for moisture, and pieces of fungus were added as needed. We use elytra length measurements as a proxy for body size (Formica et al. 2012). To measure the elytra length of each beetle, we imaged them using a flatbed scanner (Epson Perfection V600 Photo at 2100 dots per inch) and measured their elytra in ImageJ.

#### 2.2.2 Experimental Design for Laboratory Experiment

We conducted 144 no‐choice trials in total, each with a unique male and female. Seventy‐two of the males included were 1 year old, and 72 were 3 years old; past research in the lab demonstrated that a total sample size of 72 was enough to detect differences in behavior for this system (Mitchem et al. 2019), which is why we chose to test 72 males of each age class (144 total). To isolate potential effects of female age in this experiment, all females used for this experiment were 1 year old. To capture the most extreme ends of the distribution for female elytra length, we collected a total of 200 1 year old females and used the smallest 72 and largest 72 for this experiment. Our “small” 72 females (Mean elytra of 5.89 cm, SD of 0.008 cm) and “large” 72 females (Mean elytra of 6.89 cm, SD of 0.002 cm) were significantly different from one another. Large/small females were evenly divided between young and old males, after which males and females for each trial were randomly paired, and each pair was randomly assigned a trial number. In total, there were 36 assigned pairs for each trial combination: old male/large female, old male/small female, young male/large female, and young male/small female. Choice trials were conducted in 19.4 × 16.5 × 11.4 cm plastic containers containing a “2” × “2” square of *Ganoderma tsugae* as a food resource and filled the base with plaster of Paris to help retain moisture and to make a more level surface for the beetles to prevent toppling. Each male in the trial was marked with a silver paint dot using non‐toxic paint (Testor, Rockford, Illinois) to differentiate it from the female. While we cannot confirm if marking the males with paint changes their behavior, every male was marked, so any difference would be distributed equally, thus allowing for comparisons between males. Trials started with the male and female of each trial placed on opposite sides of the *G. tsugae* square and ended after 15 h of recording. After each trial, we returned beetles to their cups and housed them until the end of the summer when they were placed back into their respective breeding enclosures. New chambers were constructed for each trial.

We performed behavioral scan sampling of our mating trials to determine male preference for female elytra length. Forked fungus beetles are most active during low‐light periods of evening and early morning (Liles 1956; Pace 1967), so we video recorded choice trials in the dark using an infrared camera (Basler ace L acA4096‐30um, Ahrensburg, Germany) and infrared lights to assist with visibility. Cameras recorded one frame per second. Videos were scored by scan sampling using InqScribe software (Inquirium, Chicago, Illinois). To record behaviors, we performed instantaneous scan sampling every 5 min for the entire 15‐h trial, and pairs were scored as courting, attempting copulation, guarding, or nothing. Of these behaviors, two of them were used as proxies for mating preference: (1) courtship, when the male was head‐to‐abdomen with the female, and (2) copulation attempt, when the male is actively attempting to insert his aedeagus. We used the proportion of time in the trial spent in courtship or copulation attempts as indicators for preference, a metric commonly used in previous studies of a variety of taxa (Olsson 1993; Bonduriansky 2001; Ccouldridge and Alexander 2001).

To test for changes in male mate preference for female elytra length in our mating trials we conducted generalized linear mixed models (GLMM) in the R package glmmTMB (Brooks et al. 2017). We tested uniformity, zero inflation, and dispersion of our models using the DHARMa package in R (Hartig 2022). All models passed these assumptions unless otherwise noted. We assessed the significance of our models with type‐three Wald *χ* ^2^ test using the ‘car’ package (Fox and Weisberg 2019). The proportion of time a male spent courting was our dependent variable while male age, female body size, and their interaction were main effects; this interaction between male age and female body size would detect if male preference for female size changes with male age. Predicted values from our model were back transformed to their original values (i.e., millimeters for female body size) to allow for more straightforward comprehension of the preference gradient. Male body size was also included as a main effect in the model, as male body size is expected to play a role in male courtship behaviors and success (Formica et al. 2021). Because only 12 trials fit under the camera at a time, we recorded trials over 12 days and included the date of trial as a random effect to account for variation among recording days. For this model we used an ordered beta regression distribution.

## 3 Field Study

**FIGURE 2:**
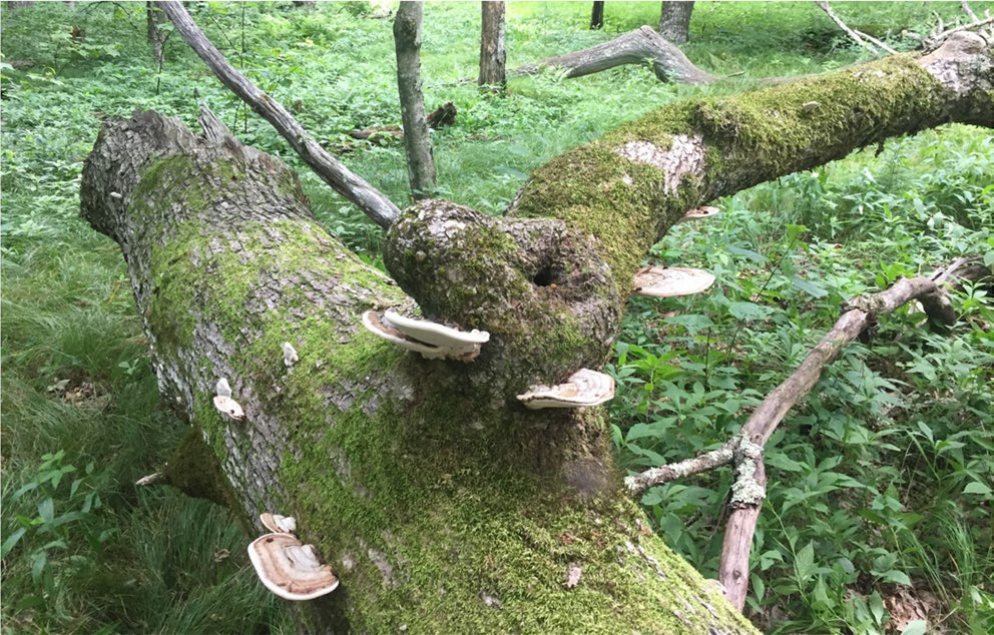
A Pond Drain subpopulation. This log in the Pond Drain region of Pembroke, VA supports multiple brackets of *Ganoderma applanatum* fungus, as well as several forked fungus beetles (dark dots on tops of fungus brackets). Picture credit belongs to Vince Formica.

Beetle ages were identified through mark‐recapture methods in which we attempted to capture all beetles in a 2 km^2^ area. We began mark‐recapture sampling in 2015, when we first mapped all logs found in the Pond Drain region that contained any of the three species of host fungus. We captured and marked all beetles found within 100 m of the 16 focal subpopulations, as 100 m is double the median dispersal distance of individuals in this system (Ludwig et al. 2008). Subpopulations are defined as the group of beetles that reside on a single log (Figure 2). Beetles on each log are socially distinct and movement between logs is minimal (Wood et al. 2018; Formica et al. 2021; Ludwig et al. 2008). Each year we surveyed every subpopulation at least three times to identify newly eclosed adults in the metapopulation. Previous work demonstrates that surveying three times a year is sufficient to capture at least 85% of all beetles present in our subpopulations (Greene et al. 2025; Formica et al. 2021). After the initial 2015 sampling, we assumed that any unlabeled individuals we encountered in 2016 had newly eclosed and were classified as 1 year old. We classified individuals that survived from 2016 to 2017 as 2‐year‐olds. As a result, beetles that were first marked in 2016 and recaptured in 2018 were deemed to be 3‐year‐olds. We may have overlooked some individuals in peripheral populations, which could have led us to underestimate their true age. This error likely diminished the observed differences among age classes.

**FIGURE 3:**
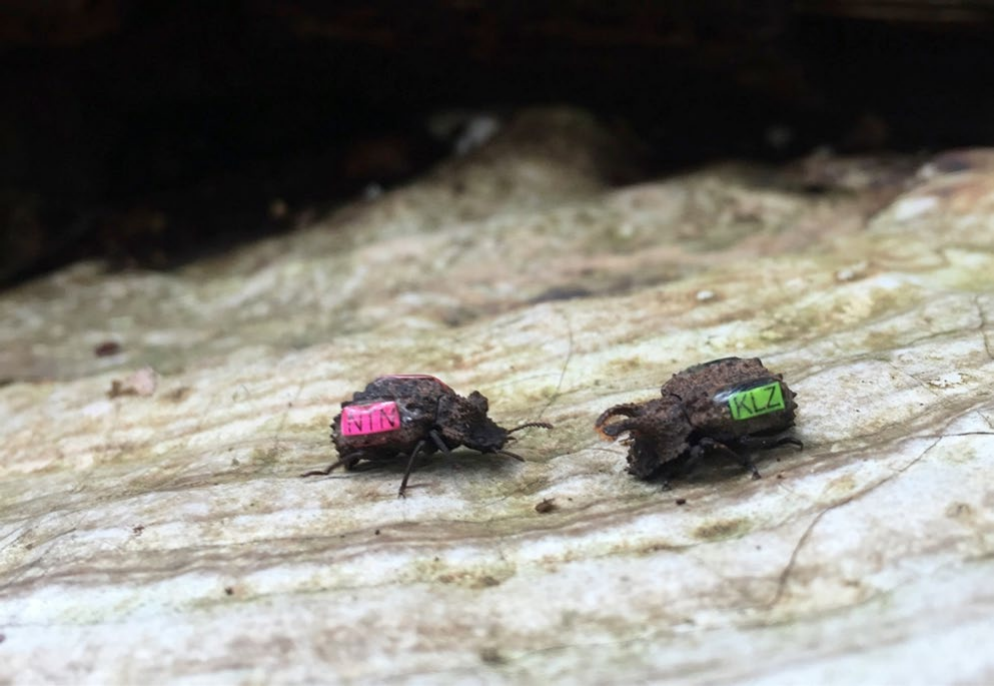
Unique fluorescent labels identify focal beetles. This female (left) and male (right) beetle were each marked with fluorescent three‐letter labels to provide easy identification during field observations. Picture credit belongs to Vince Formica.

We conducted this study at Mountain Lake Biological Station in Pembroke, VA (37.375613, −80.523176), where we have studied the Pond Drain metapopulation since 2015. Following the methods outlined in Formica et al. (2021), we captured all *B. cornutus* from the focal subpopulations. We measured elytra length to the nearest 0.01 mm from the images in ImageJ (Abràmoff et al. 2004). After elytra measurements, we applied fluorescent labels with a unique three‐letter code to both sides of their elytra using a light‐cured acrylic (Figure 3) (Tuffleye Wet‐A‐Hook Technologies). We processed and returned all individuals to the original log they were collected from within 48 h. To capture instances of male preference in the wild, we used trained observers to scan the surface of the logs and fungal brackets in every subpopulation during three scanning periods each day (06:00–11:00, 13:00–18:00, and 21:00–02:00) for about 60 consecutive days from June to August between 2016 and 2019. Observers recorded all instances of reproductive behaviors, including courtship, copulation attempts, and guarding behaviors, along with the location and individual ID of each beetle engaged in each behavior. The observers were blind to the ages of the beetles as well as the hypotheses regarding male age and female preference.

We used the same statistical infrastructure and packages as described above for our laboratory experiment. Our first goal was to perform an analysis that would confirm the presence of male mate preference in wild subpopulations as described in (Formica et al. 2016). We then wanted to determine if there was a difference among male age groups in their average mate size, indicating a potential difference in preference among male age groups. Because both males and females were observed mating with multiple partners in the wild, we conducted two separate statistical models. The first model used females as the unit of analysis and examined if the size of females influenced how often she was courted. For this model, we used the total number of times each female was observed being courted in a year as the dependent variable, with female elytra length as the main independent variable. The survey year and the total number of times each female was seen in a year were also included as fixed effect covariates known to influence variation in mating behavior and success (Cook et al. 2023). The female ID and sub‐population ID were included as random effects. This model was specified as a zero‐inflated model with a truncated Poisson distribution family.

The second model used male courtship observations as the unit of analysis and determined if the age of males predicted the size of the females he courted. Female elytra were used as the dependent variable with male age as the main independent variable. Because male size is known to play a large role in predicting courtship effort and success, we included male elytra size as a covariate fixed effect; survey year was also included as a fixed effect. Male ID and sub‐population ID were included as random effects. We used a Gaussian distribution for this model as it was the best fit to the data; while the residuals were not normally distributed (in this or any family) the residuals were not overdispersed or heteroskedastic, and the data was not zero‐inflated.

## 4 Results

### 4.1 Laboratory Experiment Results

In the lab, both young and old males spent significantly more time courting larger females (*χ* ^2^ = 6.56, *p* = 0.01; Table 1). There was also no significant effect of male age (*χ* ^2^ = 2.91, *p* = 0.09; Table 1) or male body size (elytra in mm) (*χ* ^2^ = 2.91, *p* = 0.09; Table 1). Further, we tested whether there were quadratic effects present in our dataset but found no significant relationships.

**TABLE 1:** Generalized linear mixed model (GLMM) outputs for laboratory experiments and wild observations.

Lab experiment model outputs				
Fixed effect	Estimate	*𝝌* ^2^	Df	*p*
Intercept	−1.12	0.52	1	0.47
Male elytra length	−0.33	2.91	1	0.09
Female elytra length	0.40	6.56	1	0.01
Male age	−1.72	2.91	1	0.09
Male age × female elytra length	0.25	2.52	1	0.11

Wild observation model outputs				
Fixed effect	Estimate/marginal means	*𝝌* ^2^	Df	*p*
Intercept	9.24	0.03	1	0.85
Male age	1 y.o. = 6.79 2 y.o. = 6.78 3 y.o. = 6.71	1.38	2	0.50
Male elytra length	−0.006	0.04	1	0.84
Survey year	−0.001	0.002	1	0.96

### 4.2 Field Study Results

We first confirmed the results of our laboratory experiment by assessing how often females of different sizes were courted. Males were observed courting larger females significantly more often than smaller females (*χ* ^2^ = 6.824, *p* = 0.009; Figure S1 and Table S1). As expected, the covariates of our model explained variation in mating frequency (*χ* ^2^ = 24.893, *p* < 0.001; *χ* ^2^ = 232.401, *p* < 0.001). Comparing age groups in the wild, there was no significant difference between males of different ages and their average mate size (elytra in mm) (*χ* ^2^ = 1.38, *p* = 0.50; Figure 5 and Table 1). Male size also did not significantly affect the average of his mate's size (*χ* ^2^ = 0.04, *p* = 0.84; Table 1). Similar to our laboratory experiment, no significant quadratic effects were found.

## 5 Discussion

**FIGURE 4:**
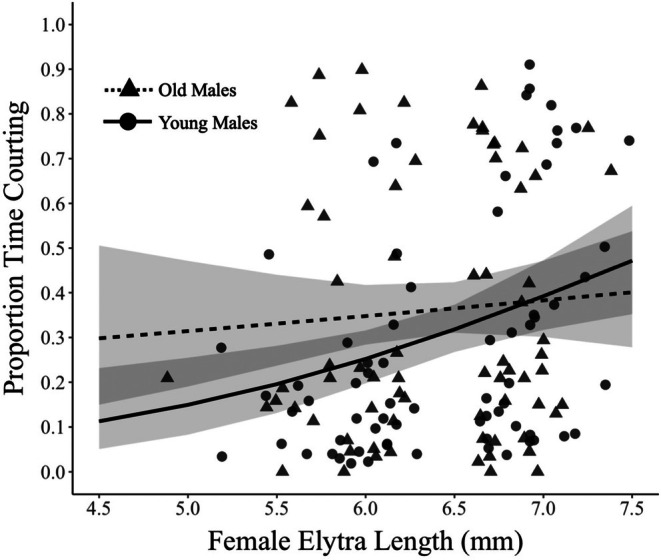
Males of different age groups did not show a difference in preference for large females. Both age groups spent significantly more time mating with larger females. However, there was no significant difference between the preference slopes of young and old males, indicating that there is no significant change in preference level as the male ages. Preference slopes and 95% CI shading predicted based on the model described in the text and back transformed to fit raw data points.

**FIGURE 5:**
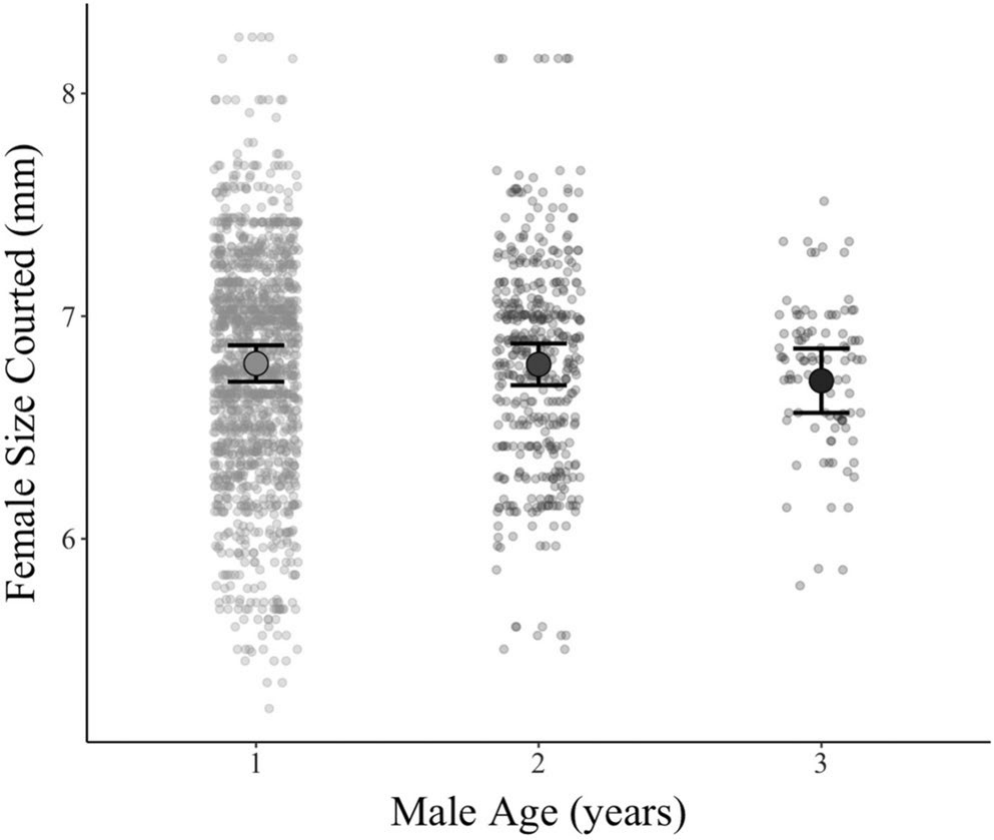
Males in the wild show no difference for average female mate size among age groups. Distribution of the average female size that each age class of males was observed courting in the wild. These observations found no significant difference between the average size of mates across male age groups. Circles and error bars show group means and 95% confidence intervals, respectively.

Our study investigated age as a potential factor influencing male mate preference in the long‐lived beetle *Bolitotherus cornutus*. We found that both wild and captive beetles displayed a preference for larger females (Table 1 and Figure S1). However, this preference did not significantly differ between young and old males (Figure 4 and Figure 5). These findings suggest that the age of the male itself may not be a significant predictor of individual variation in male mate preference—a result contrasting past findings (Rundus et al. 2015). Instead, males may be using short‐term cues, such as the upcoming end of the breeding season, to alter their mate selection; this possibility would require future studies, as our experiments were limited to the range of the summer, while the beetles remain active until October to early November (Liles 1956; Pace 1967). Our findings underscore the importance of studying phenotypic variation between age groups in systems that live for multiple breeding seasons and draw attention back to other cues that may influence male mate preference (Edward and Chapman 2011).

These results align with theoretical predictions and previous findings in male mate preference research. Male mate preference is more likely to evolve when males frequently interact with females, if the costs of mating are relatively high, and if females vary in a trait that correlates with fecundity or mating status (Edward and Chapman 2011). *B. cornutus* populations live in social groups, so males are able—if not likely—to simultaneously encounter multiple females (Conner 1989). For costs of mating, males may spend hours courting before a female accepts a copulation, after which he can spend many more hours guarding her—time that could be spent searching for other mates (Conner 1988). Finally, past work in this species has found that female body size is a predictor of female fecundity, with larger females also being more fecund (Formica et al. 2016). A caveat to consider about female quality is that we cannot confirm the mating status of each female before collection or observation. Thus, a differential preference for unmated females among age groups could present a confound when comparing overall male mate preference (Duffield et al. 2018; Richardson and Zuk 2023). This effect could emerge if *B. cornutus* unmated and mated females show similar levels of choosiness, a trend seen in past meta‐analyses (Dougherty 2023; Richardson and Zuk 2024). Asymmetry between male and female preference as it relates to female mating status would both introduce variation into male mate preference and decrease the effectiveness of using female ID as a random effect.

A fairly unique (Aich et al. 2021) feature of this beetle is its lifespan (Cook et al. 2024). In the wild, forked fungus beetles often reach 3 years of adult (i.e., post metamorphosis) age, with some individuals recorded at 5 years old (Conner 1989). By combining the high costs of mating and convenience of an insect system with an extraordinary lifespan for a beetle, *B. cornutus* becomes an ideal system to study how age may influence male mate preference. As previous work has suggested, males with preference for more fecund females must balance the costs of denying present mating opportunities with the benefits of saving for future—and potentially more productive—mating opportunities. This present‐to‐future balance model predicts that males with a finite window of breeding time are expected to decrease their level of preference as that breeding time nears its end (Engqvist and Sauer 2002; Reinhold et al. 2002; Galvani and Johnstone 1998). If we were to apply this theory to an individual's entire lifespan, it would be reasonable to expect that preference for larger females should subsequently decrease as males get older, an argument made in past empirical work (Rundus et al. 2015). However, the short lifespan of past study systems calls into question whether age itself drove this decrease or if the males were responding to a short‐term cue, such as the end of the breeding season (Nickley et al. 2016; Dondale and Binns 1977).

Our study found that males of different age classes did not significantly differ in the strength of their preference for large females (Figure 4), a contrast to our expectations and the results of past experimental work. Because these results are supported by analyses of data from our natural observations of wild subpopulations (Figure 5), we are confident that our results represent a true ecological relationship. These results also suggest that age itself may not have as significant an influence on male mate preference as more immediate temporal cues or other well‐established drivers of preference, such as how often males encounter females (Edward and Chapman 2011).

Surprisingly, old males and young males in our laboratory experiment did not differ in their total time spent courting, showing no differences in reproductive investment between age classes. This contrasts with recent empirical work in insects demonstrating that males increase their time spent mating as they age (Karl et al. 2013; Mysterud et al. 2005; Jones and Elgar 2004; Greene et al. Submitted), and we would therefore expect old males to spend longer periods of time in courtship on average in this system. This result could be due to the artificial husbandry and laboratory conditions. Males were isolated for several weeks prior to being placed in mating trials to ensure that previous mating experiences would not influence future mating behavior. However, it is possible that males perceived this period of isolation as a low female encounter rate and behaved as they would in an incredibly mate‐limited environment (Barry and Kokko 2010), potentially washing out major discernible age differences.

In the wild, old male *B. cornutus* are observed to exhibit more overall courtship effort (Greene et al. Submitted). Measuring the change in overall male courtship effort for females of differing sizes (i.e., preference) in the wild could be more effective for observing age‐based differences in overall reproductive behavior. However, the logistical requirements of such an experiment call into question its feasibility. Our current analysis of wild observations, as seen in Figure 5, has much larger sample sizes than our laboratory experiment, increasing our ability to detect age‐based differences in total reproduction investment. Even so, no difference in average mate size was detected among males of different age groups (Figure 5), suggesting that our results from our laboratory experiment carry some external validity.

Investigating the relationship between age and male mate preference requires careful consideration of a multitude of factors, where researchers must account for other established drivers of male mate preference, such as the rate a male encounters mates, the relative cost of mating, and the variation in female fecundity (Edward and Chapman 2011). Although decreasing preference as a function of age is an intuitive extension of past work focusing on breeding seasons (Engqvist and Sauer 2002; Reinhold et al. 2002; Galvani and Johnstone 1998), our results suggest that males may not be modulating their mating behavior in response to age, or that fecundity differences among females are strong enough that all males benefit from mating with larger and more fecund females. Our system also allows us to better generalize our work to longer lived species that have displayed male mate preference, such as birds (Griggio et al. 2009; Amundsen et al. 1997) and mammals (Muller et al. 2006; Craig et al. 2002). One aspect of age that should be considered is terminal investment. Research has shown that males increase their reproductive effort in response to a mortality cue, such as inoculation of heat‐killed bacteria (Velando et al. 2006; Kivleniece et al. 2010; Duffield et al. 2017; Clutton‐Brock 1984; Williams 1966). This phenomenon may have implications for male mate preference and its relationship to natural senescence. Though the rate of aging is unknown in this system, forked fungus beetles of up to 6 years old have been recorded in captivity and the wild; it may be that their senescence is rarely captured in the wild, and most mortality in the system is limited to predation or external injury (Conner 1988; Pace 1967). A slow rate of senescence could make it difficult to observe strictly age‐based terminal investment–and subsequent decrease in preference level–from occurring. Another potential explanation for our results may be that older males are compensating for any negative effects of aging on future courtship by increasing their rate of interacting with females in the present. Increasing female encounter rate would effectively balance out the decrease in future reproductive events, maintaining an equilibrium of mate preference across the lifespan. Previous work has shown that older beetles are more aggressive, and that aggression independently predicts winning fights (Greene et al. Submitted; Mitchem et al. 2019). It could be that older males, if they win more fights, increase their access to potential mates and thus negate any effects of age–further studies would be required to make this conclusion.

## Author Contributions

**Griffin M. Jiron:** conceptualization (equal), data curation (equal), formal analysis (equal), investigation (equal), methodology (equal), project administration (equal), validation (equal), visualization (equal), writing – original draft (equal), writing – review and editing (equal). **Charlotte A. Greene:** conceptualization (equal), data curation (equal), formal analysis (equal), funding acquisition (equal), investigation (equal), methodology (equal), project administration (equal), validation (equal), visualization (equal), writing – original draft (equal), writing – review and editing (equal). **Edmund D. Brodie III:** conceptualization (equal), funding acquisition (equal), investigation (equal), methodology (equal), project administration (equal), resources (equal), supervision (equal), validation (equal), writing – review and editing (equal). **Vincent A. Formica:** conceptualization (equal), data curation (equal), formal analysis (equal), funding acquisition (equal), investigation (equal), project administration (equal), supervision (equal), visualization (equal), writing – review and editing (equal).

## Ethics Statement

Our study follows all proper animal guidelines for the treatment of animals in behavioral research and teaching—ScienceDirect, and is in accordance with both animal policies at the University of Virginia. No *B. cornutus* individuals were harmed during this process.

## Conflicts of Interest

The authors declare no conflicts of interest.

## Supporting information


**Data S1:** Supporting Information.

## Data Availability

All the required data are uploaded as Supporting Information, and data will be archived in the LibraData repository at https://library.virginia.edu/libra.
