# Supplementary material for: Male Mating Preference for Larger Females Does Not Vary Among Age Classes in the Long‐Lived Beetle Bolitotherus cornutus
Source: Ecol Evol. 2025 Aug 14;15(8):e71958. doi: 10.1002/ece3.71958 (PMC12353013; doi:10.1002/ece3.71958)
Supplement: Supplementary file 1 — Data S1: Supporting Information. [file ECE3-15-e71958-s001.docx]

**Supplemental Figures**


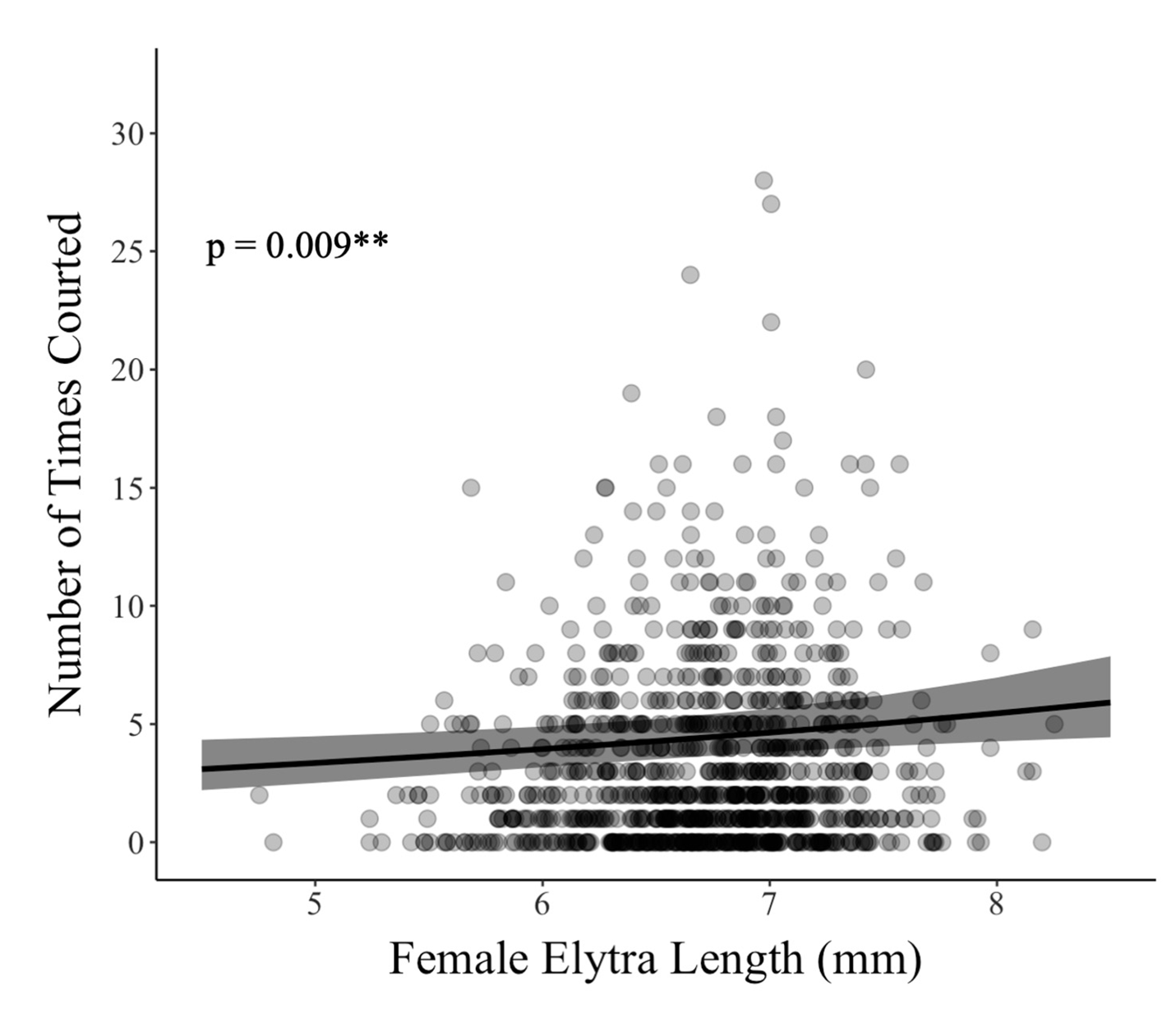


**Supplemental Figure 1: Natural observations find large females in courtship more often.** Observations taken in natural populations of *B. cornutus* found a significant relationship between female size and her rate of being courted by males, with larger females being courted more often than smaller females. There was no significant difference between the ages of the males courting these females. Preference slope and 95% CI shading back transformed from GLMM mentioned in text and Supplemental Table 1 to fit original data points.

**Supplemental Table 1: Results from GLMM predicting observed courtship events from female elytra length.**

| Fixed Effect | Estimate | 𝝌2 | Df | P value |
| --- | --- | --- | --- | --- |
| **Intercept** | 398.29 | 24.904 | 1 | <0.001 |
| **Female Elytra Length** | **0.16** | **6.824** | **1** | **0.009** |
| **Survey Year** | -0.20 | 24.893 | 1 | <0.001 |
| **Number of times observed** | 0.43 | 232.401 | 1 | <0.001 |
